# Ghrelin Attenuates Liver Fibrosis through Regulation of TGF-β1 Expression and Autophagy

**DOI:** 10.3390/ijms160921911

**Published:** 2015-09-10

**Authors:** Yuqing Mao, Shaoren Zhang, Fujun Yu, Huanqing Li, Chuanyong Guo, Xiaoming Fan

**Affiliations:** 1 Department of Gastroenterology and Hepatology, Jinshan Hospital of Fudan University, Shanghai 201508, China; E-Mails: yqmao14@fudan.edu.cn (Y.M.); 12211270005@fudan.edu.cn (S.Z.); 13111270002@fudan.edu.cn (F.Y.); 12231270002@fudan.edu.cn (H.L.); 2 Department of Gastroenterology and Hepatology, Shanghai Tenth People’s Hospital of Tongji University, Shanghai 200072, China

**Keywords:** ghrelin, CCl_4_, bile duct ligation, hepatic stellate cells, fibrosis, TGF-β1-Smad, NF-κB, autophagy

## Abstract

Ghrelin is a stomach-derived growth hormone secretagogue that promotes various physiological effects, including energy metabolism and amelioration of inflammation. The purpose of this study was to investigate the protective mechanism of ghrelin against liver fibrosis. Liver fibrosis was induced in C57BL/6 mice by intraperitoneal injection of CCl_4_ (2.0 mL/kg of 10% CCl_4_ *v*/*v* solution in peanut oil) two times per week for eight weeks. Ghrelin (10 μg/kg) was intraperitoneally injected two times per week for eight weeks. A second murine liver fibrosis model was induced by bile duct ligation (BDL) and concurrent ghrelin administration for four weeks. Hematoxylin eosin (H&E), and Masson’s trichrome were used to detect pathological changes to liver tissue. Western blotting was used to detect protein levels of transforming growth factor (TGF)-β1, phosphorylated Smad3 (p-Smad3), I-collage, α-smooth muscle actin (α-SMA), matrix metalloproteinases (MMPs) 2, tissue inhibitor of matrix metalloproteinases (TIMPs) 1, phosphorylated NF-κB (p-NF-κB), and microtubule-associated protein light chain 3 (LC3). In addition, qRT-PCR was used to detect mRNA levels of TGF-β1, I-collage, α-SMA, MMP2, TIMP1 and LC3, while levels of TGF-β1, p-Smad3, I-collage, α-SMA, and LC3 were detected immunohistochemically. Levels of aspartate aminotransferase and alanine aminotransferase were significantly decreased by ghrelin treatment. Ghrelin administration also significantly reduced the extent of pathological changes in both murine liver fibrosis models. Expression levels of I-collage and α-SMA in both models were clearly reduced by ghrelin administration. Furthermore, ghrelin treatment decreased protein expression of TGF-β1 and p-Smad3. The protein levels of NF-κB and LC3 were increased in the CCl_4_- and BDL-treatment groups but were significantly reduced following ghrelin treatment. In addition, ghrelin inhibited extracellular matrix formation by decreasing NF-κB expression and maintaining the balance between MMP2 and TIMP1. Our results demonstrated that ghrelin attenuates liver fibrosis via inhibition of the TGF-β1/Smad3 and NF-κB signaling pathways, as well as autophagy suppression.

## 1. Introduction

Liver fibrosis is a wound-healing response that ultimately results in the progressive accumulation of an extracellular matrix (ECM) [1,2]. However, this process is also a necessary stage for progression of many chronic liver diseases to cirrhosis and even liver carcinoma [3]. Hepatic fibrosis is among the leading causes of morbidity and mortality worldwide and results from several well-known risk factors, such as viral infection, drug use, autoimmune imbalances, alcohol abuse, and metabolic disorders [4]. However, effective pharmaceutical therapies are still lacking. Therefore, the design of novel effective drugs and strategies for the treatment of hepatic fibrosis is urgent.

Under normal conditions, hepatocytes have a remarkable regenerative capacity, and fibrotic cells are quiescent; however, this changes when hepatocytes suffer heavy and sustained damage. Among activated fibrotic cells, hepatic stellate cells (HSCs) are the most important in the progression of liver fibrosis. Once quiescent HSCs become activated, they transform into proliferative and contractile myofibroblast-like cells, which produce abundant collagen I, the major component of ECMs [5]. Other related structural proteins and receptors are also produced by activated HSCs, including α-SMA and the platelet-derived growth factor (PDGF) receptor [6,7]. Furthermore, Kupffer cells and other inflammatory cells produce cytokines, which play important roles in the activation of HSCs, with TGF-β1 being the most critical. Activated HSCs also express the TGF-β1 receptor to maintain continuous activation [8,9,10,11]. Liver fibrosis is the product of the imbalance between ECM production and degradation, which is mediated by two important proteins produced by HSCs: matrix metalloproteinases (MMPs), which promote ECM degradation, and TIMPs, which contribute to ECM formation. Both MMP2 and TIMP1 regulate the process of liver fibrosis via TGF-β1 mediation [12].

As described above, hepatic fibrosis is initiated by hepatocyte damage, which leads to recruitment of inflammatory and Kuppfer cells, and the subsequent release of cytokines, which, in turn, promotes HSC activation [13]. Therefore, inflammation is the initiator of fibrosis in a process governed by the NF-κB signaling pathway. Hence, this pathway presents a critical target to ameliorate inflammation and liver injury in order to lessen the extent and severity of liver fibrosis [14].

Autophagy is a catabolic intracellular pathway, targeting defective organelles and excessive components to the lysosomes for degradation [15], usually activated by energy restriction, stress, or inflammation. There are three different kinds of autophagy: macroautophagy, microautophagy, and chaperone-mediated autophagy [16]. LC3II is located on the autophagosome membrane and is widely used as a marker to monitor autophagy. Furthermore, the protein beclin-1 also participates in the process of autophagy [17]. Recent studies have demonstrated that autophagy is closely associated with liver fibrosis. For example, Thoen *et al.* [18] discovered that autophagic flux was increased during HSC activation but was inhibited by bafilomycin A1, an autophagy inhibitor. Hernandez-Gea *et al.* [19] reported that autophagy releases lipids from intracellular lipid droplets, which then promotes fibrogenesis via activation of HSCs in both mouse and human tissues. Therefore, autophagy fuels HSC activation and may be a promising therapeutic target for the treatment of liver fibrosis.

Ghrelin is a peptide that was first purified from the stomach of rats and has been shown to be a natural ligand of the growth hormone secretagogue (GHS) receptor type 1a (GHS-R1a). There are two forms of ghrelin, acylated and unacylated, with the previous form usually regarded as biologically active [20]. Although mostly produced in the stomach, ghrelin transcripts have been detected in many other organs, including the liver, heart, bowel, pancreas, kidneys, thyroid, and lungs [21]. Described as an energy regulator, ghrelin was demonstrated to promote appetite, control energy expenditure, and ameliorate cancer and heart failure-induced cachexia [22]. In addition, recent studies have shown that ghrelin exhibited various biological effects, such as inhibition of inflammation and apoptosis. Therefore, ghrelin administration might suppress the proliferation of immune cells and the release of inflammatory cytokines [23,24,25,26]. Ghrelin also contributes to protection against hepatocellular injury induced by ischemia/reperfusion [27]. Moreno *et al.* [28] first demonstrated that ghrelin reduced hepatic fibrosis by increasing hepatic expression of hepatoprotective signaling pathways such as phospho-Akt (p-Akt) and phospho-extracellular signal-regulated kinase (p-ERK). Although these results are encouraging, the other anti-fibrosis mechanisms of ghrelin are not totally clear and it is uncertain whether autophagy is involved in ghrelin-induced fibrosis reduction.

In this study, we provided evidence that acylated ghrelin exerts antifibrotic effects against liver fibrosis via regulation of the TGF-β1/Smad3 signaling pathway. Ghrelin administration also inhibited the NF-κB pathway and maintained the balance between MMP2 and TIMP1. We also demonstrated that ghrelin reduced fibrosis via autophagy inhibition, which may limit the available energy for activation of HSCs.

## 2. Results

### 2.1. Liver Fibrosis Was Reduced by Ghrelin Administration in Both CCl_4_- and BDL-Induced Liver Fibrosis Mouse Models

**Figure 1:**
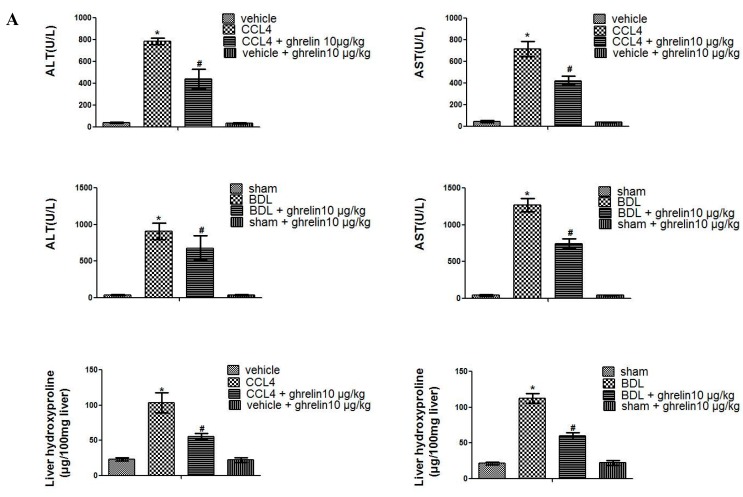

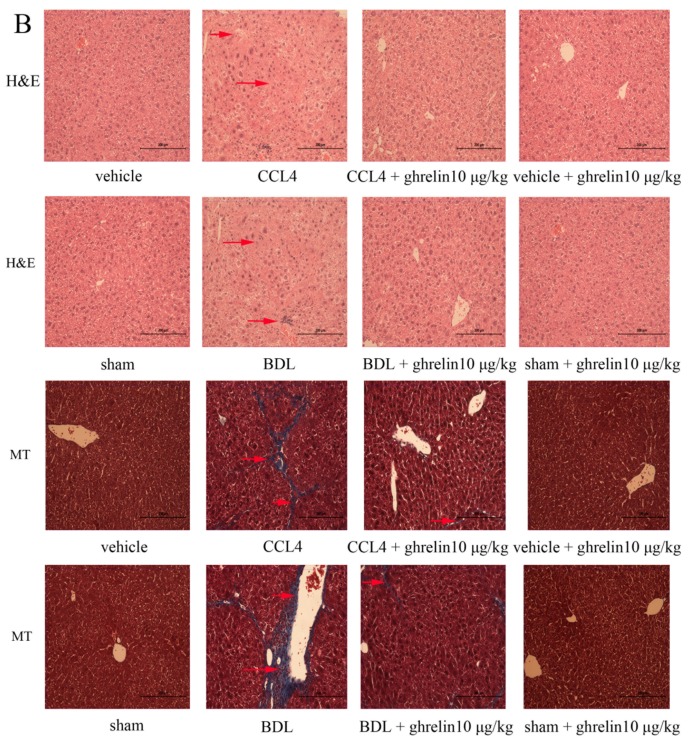
Effect of ghrelin on CCl_4_- and BDL-induced liver fibrosis. (**A**) Ghrelin decreased the levels of ALT, AST, and hydroxyproline. Data are expressed as the mean ± SD (*n* = 7, * *p* < 0.05 for CCl_4_ or BDL group *vs.* the vehicle or sham group, # *p* < 0.05 for CCl_4_ + ghrelin or BDL + ghrelin group *vs.* the CCl_4_ or BDL groups); and (**B**) Ghrelin ameliorated pathological changes to the liver, as demonstrated by H & E and Masson’s trichrome (MT) staining (original magnification: 200×). Red arrows indicate damaged liver tissue and fiber cords. BDL: bile duct ligation; ALT: alanine transaminase; AST: aspartate transaminase; H & E: hematoxylin and eosin; MT: Masson’s trichrome. The scale bar of each figure is 200 μm, and the magnification is 200×.

We first detected serum levels of alanine transaminase (ALT) and aspartate transaminase (AST) to evaluate the effect of ghrelin administration on liver injury. As shown in Figure 1A, serum levels of ALT and AST were significantly increased in both CCl_4_- and BDL-treatment groups, as compared with the control group. Ghrelin treatment obviously decreased ALT and AST levels, indicating that co-treatment significantly attenuated CCl_4_- and BDL-induced hepatocyte injury. Furthermore, ghrelin treatment significantly reduced the CCl_4_- or BDL-induced increase in hydroxyproline content, which is a known marker of total collagen content. H & E staining revealed severe pathological changes in liver sections from both CCl_4_ and BDL groups, as evidenced by damage or death of hepatocyte, and rearrangement of liver lobular structures, and the formation of pericellular bridging fibrosis. These areas of damaged tissues were markedly reduced in liver sections collected from the ghrelin-treated group (Figure 1B). Collagen around the extracellular spaces, especially in the portal triad, was significantly increased in liver sections from both groups. The lobules were surrounded by bundles of blue collagen fibers; however, these were markedly reduced in the ghrelin co-treated group as compared to the CCl_4_- and BDL-treatment groups (Figure 1B). Ghrelin administration caused no changes, as observed by pathological staining.

### 2.2. Ghrelin Inhibits Activation of HSCs in Liver Fibrosis and Regulates the Balance of MMP2 and TIPM1

**Figure 2:**
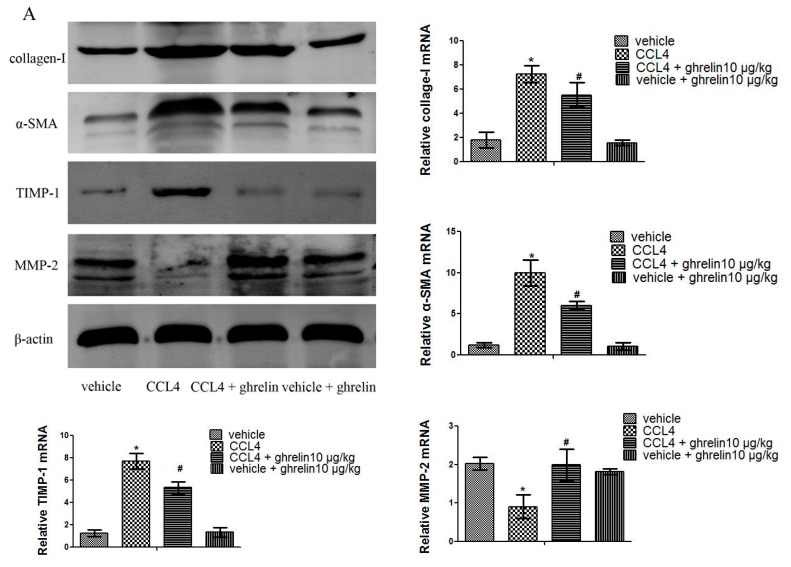

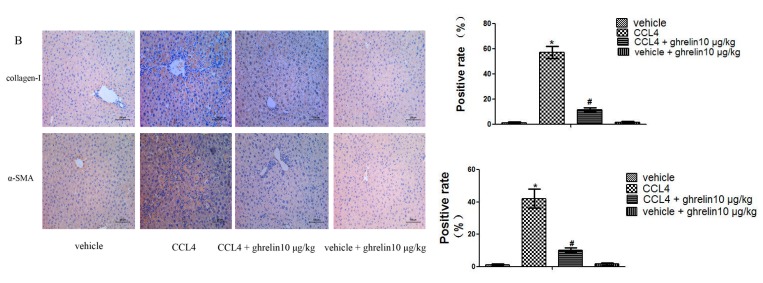
Ghrelin inhibited activation of HSCs and expression of MMP2 and TIMP1 in CCl_4_-induced liver fibrosis. (**A**) Results of Western blot and real-time PCR analyses of collagen I, α-SMA, TIMP1, and MMP2 in the CCl_4_ group; and (**B**) Immunohistochemical staining (200×) showed increased expression of collagen I and α-SMA proteins in CCl_4_-induced fibrotic liver tissues. The positive rate was analyzed with Image-Pro Plus 6.0. Data are expressed as the mean ± SD (*n* = 7, * *p* < 0.05 for CCl_4_ *vs.* vehicle group, # *p* < 0.05 for ghrelin + CCl_4_ *vs.* CCl_4_ group). HSCs: hepatic stellate cells; MMP: matrix metalloproteinase; TIMP: tissue inhibitor of matrix metalloproteinase; α-SMA: α-smooth muscle actin. The scale bar of each figure is 200 μm, and the magnification is 200×.

**Figure 3:**
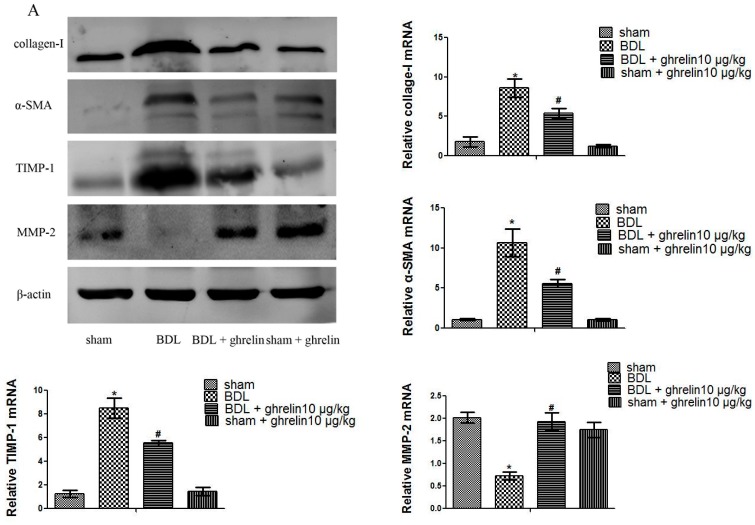

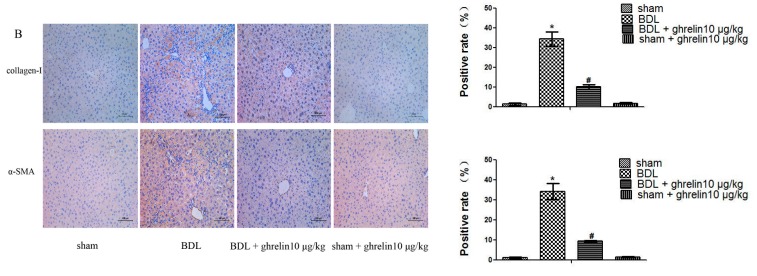
Ghrelin inhibited activation of HSCs and expression of MMP2 and TIMP1 in BDL-induced liver fibrosis. (**A**) Western blot and real-time PCR analyses of collagen I, α-SMA, TIMP1, and MMP2 in the CCl_4_ group; and (**B**) Immunohistochemical staining (200×) of collagen I and α-SMA protein in BDL-induced fibrotic liver tissues. The positive rate was analyzed with Image-Pro Plus 6.0. Data are expressed as the mean ± SD (*n* = 7, * *p* < 0.05 for the BDL *vs.* sham group, # *p* < 0.05 for the ghrelin + BDL *vs.* BDL group). The scale bar of each figure is 200 μm, and the magnification is 200×.

**Figure 4:**
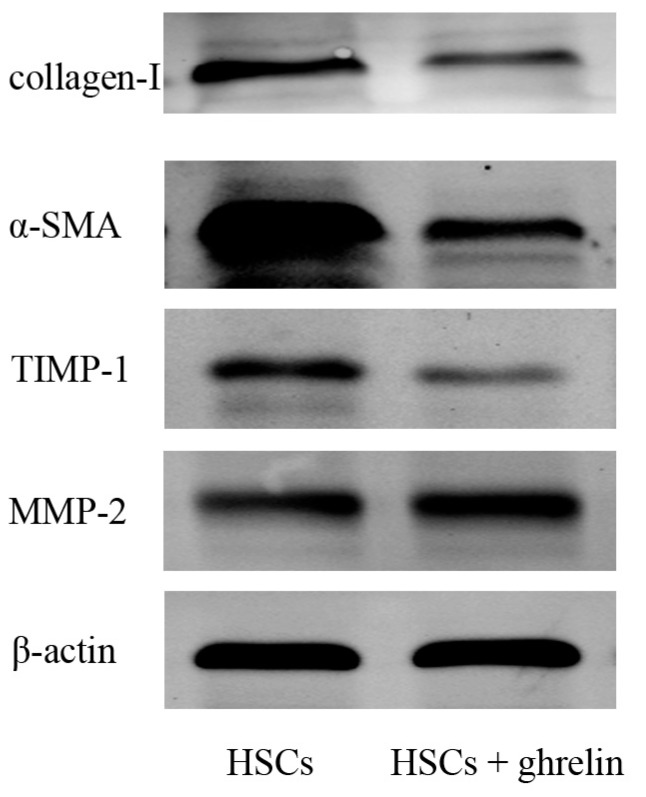
Ghrelin inhibited activation of HSCs and expression of MMP2 and TIMP1. Western blot analysis of collagen I, α-SMA, TIMP1 and MMP2.

As is shown in Figure 2A and Figure 3A, protein levels of collage I and α-SMA were significantly increased in the CCl_4_- and BDL-treatment groups, as compared with the vehicle and sham groups, while ghrelin administration reduced the expression of collage I and α-SMA. The mRNA expression levels of collagen I and α-SMA were also significantly reduced in the CCl_4_ + ghrelin and BDL + ghrelin groups. Moreover, we further detected protein levels of collagen I and α-SMA by immunohistochemical staining. Similar to the results of Western blot and qPCR analyses, the areas positive for α-SMA and collagen I were obviously reduced by ghrelin treatment, as analyzed with Image-Pro Plus 6.0 (Figure 2B and Figure 3B). The administration of ghrelin alone caused no change in the expression levels of any indicators, reflecting the activation of HSCs. We further observed that ghrelin treatment increased the expression of MMP2, which was reduced in the CCl_4_ and BDL groups. Conversely, TIMP1 expression was obviously increased in both groups and also was reduced by ghrelin treatment at both the mRNA and protein levels (Figure 2A and Figure 3A). Similar results were observed in an *in vitro* experiment (Figure 4). These results showed that ghrelin inhibited the activation of HSCs by promoting the expression of MMP2, which plays a significant role in the cleavage of the fibrillar ECM, while decreasing levels of TIMP1, the major inhibitor of MMPs, which promotes hepatic fibrosis.

### 2.3. Ghrelin Regulates the TGF-β1/Smad3 Signaling Pathway in Liver Fibrosis

**Figure 5:**
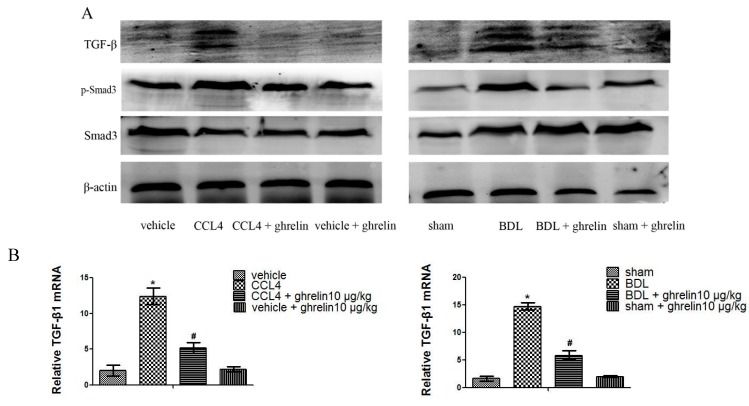

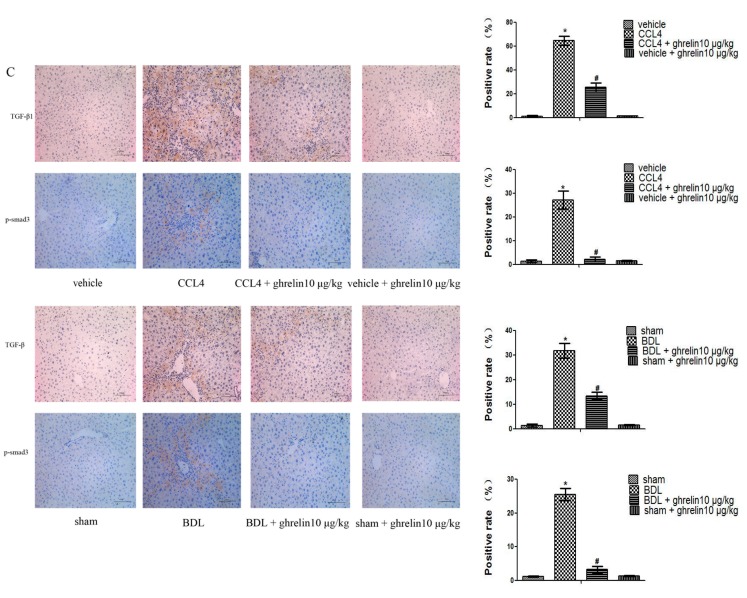
Ghrelin regulates the TGF-β1/Smad3 signaling pathway. (**A**) Western blot analysis of TGF-β1, p-Smad3, and Smad3 in the CCl_4_ and BDL fibrotic mice models; (**B**) Real-time PCR analysis of TGF-β1 is shown and (**C**) Immunohistochemical staining (200×) of TGF-β1and p-Smad3 in both CCl_4_ and BDL-induced fibrotic liver tissues. The positive rate was analyzed with Image-Pro Plus 6.0. (*n* = 7, * *p* < 0.05 for the CCl_4_ or BDL group *vs.* the vehicle or sham group, # *p* < 0.05 for the CCl_4_ or BDL + ghrelin group *vs.* the CCl_4_ or BDL group). The scale bar of each figure is 200 μm, and the magnification is 200×.

We next measured protein levels of TGF-β1, which is the most well-researched cytokine associated with liver fibrosis. Ghrelin treatment significantly decreased protein levels of TGF-β1 and p-Smad3, without affecting the total level of Smad3 (Figure 5A). TGF-β1 mRNA expression was similarly reduced in the CCl_4_ + ghrelin and BDL + ghrelin groups (Figure 5B). In addition, we found smaller positive areas of TGF-β1 and p-smad3 in the CCl_4_ + ghrelin and BDL + ghrelin groups, as compared with the CCl_4_ and BDL groups by immunohistochemical staining and as analyzed with Image-Pro Plus 6.0 (Figure 5C). These results strongly indicate that ghrelin may affect the expression of TGF-β1/Smad3 signaling pathway.

### 2.4. Ghrelin Attenuates Liver Injury via Inhibition of the NF-κB Signaling Pathway in Liver Fibrosis

**Figure 6:**
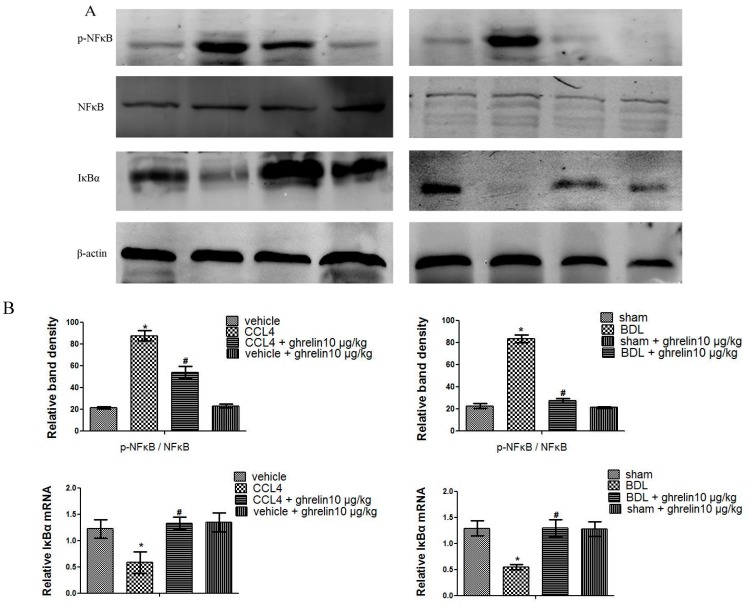
Ghrelin may be associated with NF-κB signaling pathway inhibition. (**A**) Western blot analysis of p-NF-κB, NF-κB, and IκBα in CCl_4_- and BDL-induced fibrotic liver tissues; and (**B**) Relative band density of p-NF-κB/NF-κB and real-time PCR evaluation of IκBα in both CCl_4_- and BDL-induced fibrotic liver tissues. (*n* = 7, * *p* < 0.05 for the CCl_4_ or BDL group *vs.* the vehicle or sham group, # *p* < 0.05 for the ghrelin + CCl_4_ or BDL group *vs.* the CCl_4_ or BDL group).

As shown in Figure 6, ghrelin administration suppressed p-NF-κB expression without affecting the total level of NF-κB, and increased the expression of IκBα, which was impaired in the CCl_4_ and BDL groups (Figure 6). Hence, these results indicate that ghrelin might attenuate liver fibrosis via inhibition of the NF-κB signaling pathway.

### 2.5. Ghrelin Inhibits the Autophagy Process in Liver Fibrosis

**Figure 7:**
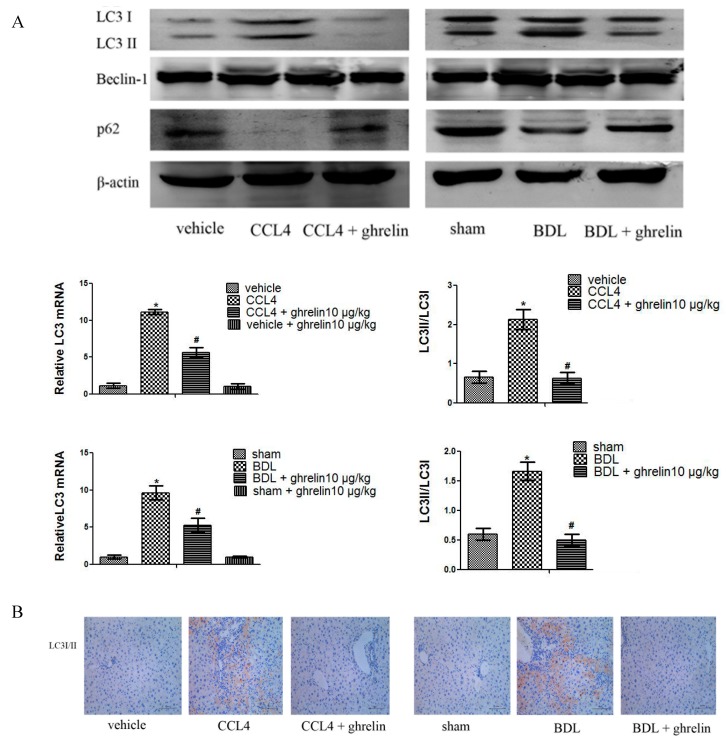
Ghrelin inhibited the process of autophagy in liver fibrosis. (**A**) Western blot analyses of LC3I/II, Beclin-1 and p62 in both the CCl_4_- and BDL-induced fibrotic liver tissues. Real-time PCR shows gene expression of LC3 (*n* = 7, * *p* < 0.05 for the CCl_4_ or BDL group *vs.* the vehicle or sham group, # *p* < 0.05 for the ghrelin + CCl_4_ or BDL group *vs.* the CCl_4_ or BDL group); and (**B**) Immunohistochemical staining (200×) of LC3I/II in the CCl_4_- and BDL-induced fibrotic liver tissues. The scale bar of each figure is 200 μm, and the magnification is 200×.

Expression of LC3 at both the mRNA and protein levels was reduced in the CCl_4_ + ghrelin and BDL + ghrelin groups, as compared to the fibrosis model groups (Figure 7A). A similar result was obtained by immunohistochemical staining (Figure 7B). However, the protein level of beclin-1 was not obviously affected by ghrelin treatment (Figure 7A). Based on this result, we suspect that beclin-1 might not participate in the process of ghrelin-induced autophagy inhibition. Furthermore, protein of p62 was reduced in fibrotic model group but significantly increased by ghrelin treatment (Figure 7A). These results indicated that ghrelin administration might inhibit the autophagy process in liver fibrosis.

## 3. Discussion

Ghrelin is a brain-gut peptide that exerts a variety of pleiotropic effects and plays critical roles in energy homeostasis and cellular protection. Amazingly, ghrelin was showed to exert obvious antifibrotic effect [28]. Another study showed ghrelin prevented doxorubicin-induced myocardial fibrosis and apoptosis via the GHSR-independent pathway [29]. The results of our research provide further evidence that ghrelin alleviates liver fibrosis through modulation of the TGF-β1/Smad3 signaling pathway and autophagy inhibition in both CCl_4_- and BDL-induced fibrosis mouse models. Although this study is not the first to demonstrate these anti-fibrotic effects, the underlying mechanisms of the anti-fibrotic mechanism of ghrelin and its relationship to autophagyis investigated in our study.

The results of the present study confirmed that acylated ghrelin administration significantly attenuated CCl_4_- and BDL-induced liver fibrosis and injury. Serum levels of ALT and AST were significantly increased in the CCl_4_- and BDL-treatment only groups but reduced by the addition of ghrelin treatment. Consistently, pathological detection of several hepatic markers by H&E and MT staining showed that ghrelin attenuated CCl_4_- and BDL-induced fibrosis, as well as maintained complete cellular structures. During chronic liver injury, HSCs lose their intracellular lipid droplets and transdifferentiate from quiescent cells to myofibroblast-like cells that have an increased proliferation rate and high production of ECM including collagen I and α-SMA. Quiescent HSCs express α-SMA at minimal levels. Our results showed that collagen I and α-SMA were highly expressed in the CCl_4_ and BDL groups but reduced by ghrelin treatment. However, the mechanism underlying ghrelin-induced inhibition of the activation of HSCs remains unknown. TGF-β1 is considered a major fibrotic mediator produced by hepatic sinus endothelial cells, Kupffer cells, activated HSCs, as well as other inflammatory cells [30]. Quiescent HSCs are activated by the stimulation of TGF-β1 in response or chronic liver injury and then secrete increased amounts of TGF-β1 to maintain continuous activation, which then induces fibrosis. Cheng *et al.* [31] demonstrated that TGF-β1 knockout with small interfering RNAs in liver stellate cells (HSC-T6) significantly decreased α-SMA and collagen I levels, as well as inflammatory cytokines IL-1 and TNF α levels, indicating that TGF-sequence-specific TGF-β1 gene silencing has potential therapeutic usefulness for the treatment of liver fibrosis. Furthermore, TGF-β1 induces phosphorylation and nuclear translocation of Smad2 primarily in quiescent HSCs and Smad3 in activated HSCs. Recent studies have confirmed that the TGF-β1/Smad3 pathway was involved in increased deposition of fibronectin, collagen I, and α-SMA in liver fibrosis [31,32]. In order to determine whether the TGF-β1/Smad3 signaling pathway was involved in the anti-fibrosis mechanism of ghrelin, we detected changes in TGF-β1 and p-Smad3 expression levels in response to ghrelin administration. As expected, ghrelin significantly reduced the CCl_4_- or BDL-induced increase in TGF-β1 and p-Smad3 expression, which indicated that ghrelin treatment reduced fibrosis at least partly via disruption of the TGF-β1/Smad3 signaling pathway. In addition, fibrogenesis is counterbalanced by fibrolysis of proteolytic enzymes, such as MMPs, while chronic damage usually favors fibrogenesis with an upregulation in TIMPs [33]. Activated HSCs express a combination of MMPs and their TIMPs, and switch the TIMP/MMP balance. As a result, excess hepatic ECM produced by myofibroblasts accumulates and leads to fibrosis [34]. We found that ghrelin increased the expression of MMP2, while decreasing levels of TIMPs, the inhibitors of metalloproteinases, as compared with the CCl_4_ and BDL treatment fibrosis model groups. These results indicated that ghrelin treatment attenuated liver fibrosis partly by reversing the imbalance in the MMP2/TIMP1 ratio induced by activation of HSCs.

NF-κB is a transcriptional regulator of genes involved in the immune and inflammatory responses [14]. IκBα is an inhibitory protein that prevents the translocation of NF-κB into the nucleus. Inflammation-induced liver injury initiates activation of myofibroblasts and subsequent ECM deposition [4]. Ghrelin has been demonstrated to modulate inflammation via inhibition of the NF-κB pathway [23]. In the present study, we observed that ghrelin inhibited the translocation of NF-κB into the nucleus, which was induced by CCl_4_ or BDL treatment, while increasing IκBα protein levels. We suspected that ghrelin ameliorated CCl_4_- and BDL-induced liver injury via downregulation of the NF-κB pathway. Furthermore, it was reasonable that this downregulation of NF-κB signaling was associated with the reduction in TGF-β1 levels produced by injured hepatic sinus endothelial cells, Kupffer cells, activated HSCs, as well as other inflammatory cells.

Autophagy is a newly recognized player in the activation of HSCs and subsequent formation of liver fibrosis [18]. Regarded as a double-edged sword [35], autophagy also plays dual roles in liver fibrosis. Autophagy may reduce fibrosis by attenuating cellular injury and energy homeostasis. However, autophagy may also supply energy for activation of HSCs by delivering triglycerides and other components f. Our results showed that LC3 expression was reduced by ghrelin treatment at both the mRNA and protein levels, as compared to treatment with CCl_4_ or BDL. It is believed that p62 accumulates when autophagy is inhibited and is decreased when there is autophagic flux. Therefore, we further detected the protein level of P62. As expected, P62 was increased by ghrelin treatment compared with CCl_4_ or BDL group, although the relationship between ghrelin and autophagy is under controversy. Some recent studies showed ghrelin stimulates autophagy [36,37,38]. However, we suspected that ghrelin might inhibit activation of HSCs via downregulation of autophagy, thus reducing available energy from intracellular lipid degradation. We speculated that the different effect of ghrelin on autophagy may due to the different experiment condition, drug doses and cellular physiological states. In addition, a recent study demonstrated that TGF-β1 may stimulate autophagy via the TGF-β-activated kinase (TAK) 1-MAPK kinase (MKK) 3-p38 and TAK1-AMP-activated protein kinase (AMPK) pathways, leading to fibrotic responses [39]. We propose that ghrelin-induced TGF-β1 inhibition might be associated with autophagy suppression and fibrosis reduction.

In conclusion, this research was the first to identify these potential anti-fibrotic mechanisms of ghrelin. We found that ghrelin treatment ameliorated CCl_4_- and BDL-induced liver fibrosis via inhibition of the TGFβ1/Smad3 and NF-κB pathways, as well as autophagy suppression. Together, these results indicate that energy regulators, such as ghrelin, offer further insight into the development of drugs for the prevention and treatment of liver fibrosis.

## 4. Experimental Section

### 4.1. Study Approval

The study protocol was approved by the Animal Care and Use Committee of Shanghai Fudan University (Shanghai, China).

### 4.2. Reagents

Acylated ghrelin was purchased from ProSpec-Tany TechnoGene, Ltd. (Hamerkaz, Israel). Carbon tetrachloride (CCl_4_) was obtained from China Sinopharm International Corporation (Shanghai, China). Antibodies against collagen I, beclin-1, MMP2, TIMP1, α-SMA, NF-κB, p-NF-κB, IκBα and p62 were purchased from Proteintech (Chicago, IL, USA). Antibodies against TGF-β1, Smad3, and p-Smad3 were purchased from Abcam (Cambridge, MA, USA). Antibody against LC3 was purchased from Cell Signaling Technologies (Beverly, MA, USA).

### 4.3. Animals and Treatment

Male C57BL/6 mice (8 weeks old, 23 ± 3 g) were purchased from Shanghai Laboratory Animal Co., Ltd. (Shanghai, China) and housed in an animal care facility under controlled light-dark cycles with constant temperature (25 ± 1 °C) and humidity (50% ± 5%), and fed a standard diet with free access to water. For CCl_4_-induced fibrosis: mice were randomly divided into four groups of seven mice each: (1) Vehicle group: mice were treated with peanut oil only; (2) CCl_4_ group: mice received an intraperitoneal injection of CCl_4_ (2.0 mL/kg of 10% CCl_4_ *v*/*v* solution in peanut oil) two times per week for 8 weeks; (3) CCl_4_ + ghrelin group: mice were injected with CCl_4_ as described above with an additional intraperitoneal injection of 10 μg/kg ghrelin two times per week for 8 weeks; (4) Vehicle + ghrelin group: mice were treated with oil and ghrelin as described above. For bile duct ligation (BDL)-induced fibrosis, mice were also randomly divided into four groups of seven mice each: (1) Sham operated group; (2) BDL group: mice underwent BDL; (3) BDL + ghrelin group: mice underwent BDL and received intraperitoneal injections of ghrelin (10 μg/kg) two times per week for 4 weeks; (4) Sham + ghrelin group: mice underwent sham surgery and received intraperitoneal injections of ghrelin (10 μg/kg) for 4 weeks. Liver and blood samples were collected for histological and molecular analyses.

### 4.4. Cell Culture and Treatment

HSC cell line was purchased from Chinese Academy of Science Committee Type Culture Collection Cell Bank. Cells were cultured in high glucose Dulbecco’s modified Eagle’s medium (Giboco, New York, NY, USA) supplemented with 10% fetal bovine serum (Hyclone, Logan, UT, USA) and 1% penicillin-streptomycin (Gibco, New York, NY, USA) in a humidified incubator at 37 °C in 5% CO_2_. We designed two groups as HSCs and HSCs + ghrelin group. 10^−8^ M ghrelin was treated with HSCs and after 24 h, cells were collected for Western blotting.

### 4.5. Biochemical Analysis

Serum was separated from blood that was collected from each mouse. Serum levels of alanine transaminase (ALT) and aspartate transaminase (AST) were measured using microplate test kits purchased from Nanjing Jiancheng Bioengineering Institute (Jiangsu, Nanjing, China) and hepatic hydroxyproline levels were measured using a commercial kit purchased from BioCheck, Inc. (Foster City, CA, USA), according to manufacturers’ instructions.

### 4.6. Histopathological Analysis

Tissue from the middle portion of the left lobe of the liver from each mouse was fixed in 4% paraformaldehyde for at least 24 h and then embedded in paraffin, from which 5 μm-thick sections were prepared and stained with hematoxylin and eosin (H & E), and Masson’s trichrome (MT) to observe tissue damage by light microscopy.

### 4.7. Immunohistochemical Analysis

The liver sections (4 μm) were dewaxed, rehydrated, and pretreated via a heat-induced antigen retrieval technique. Nonspecific sites of the tissue sections were blocked with 10% goat serum for 30 min at room temperature and then incubated overnight at 4 °C with antibodies against collage I, α-SMA, MMP2, TIMP1, LC3, p-NF-κB, TGF-β1, and p-Smad3 at dilutions of 1:500 each, and with a secondary antibody diluted at 1:50 for 60 min at room temperature. Each antibody was diluted in Tris-buffered saline supplemented with 2% bovine serum albumin. Finally, the slides were counterstained with H & E and observed under a light microscope. Three different fields of vision were randomly chosen per slide and the ratios of positive areas to total areas were acquired using Image-Pro Plus 6.0 imaging software (Media Cybernetics, Inc., Rockville, MD, USA). The average of these three ratios was used for statistical analysis. This method was applied to all groups.

### 4.8. Western Blot Analysis

Liver tissues were obtained and lysed with radioimmunoprecipitation assay buffer and protease inhibitors. Protein concentrations were measured using the bicinchoninic acid assay. HSCs were washed twice with phosphate-buffered saline (PBS) solution and lysed with RIPA buffer. Equal amounts of total protein were separated by sodium dodecyl sulfate polyacrylamide gel electrophoresis and transferred to polyvinylidene difluoride membranes, which were incubated in blocking buffer 5% nonfat milk powder dissolved in phosphate-buffered saline (PBS) for 1 h and incubated at 4 °C overnight with specific primary antibodies against collage I (dilution, 1:500), α-SMA (1:500), MMP2 (1:500), TIMP1 (1:500), LC3 (1:500), beclin-1 (1:1000), p62 (1:50), p-NF-κB (1:500), NF-κB (1:500), TGF-β1 (1:500), p-Smad3 (1:500), Smad3 (1:1000), and β-actin (1:1000). All membranes were washed with PBS + 1% Tween (PBST) and incubated with a secondary antibody (1:1000) dissolved in PBST for 1 h at room temperature. Finally, the membranes were washed three times with PBST for 10 min each and fluorescence of the reactions was detected using the Odyssey two-color infrared laser imaging system (LI-COR Biosciences, Lincoln, NE, USA).

### 4.9. RNA Isolation and Real-Time Quantitative Reverse Transcriptase Polymerase Chain Reaction (qRT-PCR)

Total RNA was extracted from frozen liver tissues with TRIzol reagent (Tiangen Biotech Co., Ltd., Beijing, China) and reverse-transcribed using the Reverse Transcription Kit (TaKaRa Biotechnology Co., Ltd., Dalian, China). The resulting cDNA was used as the template for quantitative RT-PCR with primers specific for collage I, α-SMA, MMP2, TIMP1, LC3, TGF-β1, and β-Actin (see Table 1) according to the instructions of the SYBR Premix EX Taq kit (TaKaRa Biotechnology Co., Ltd.) and using the 7900HT Fast Real-Time PCR system (Applied Biosystems, Foster City, CA, USA).

**Table 1:** Nucleotide sequences of primers used for PCR.

Gene	Primer Sequence (5′-3′)	
Collagen I	Forward	CAATGGCACGGCTGTGTGCG
	Reverse	AGCACTCGCCCTCCCGTCTT
α-SMA	Forward	CCCAGACATCAGGGAGTAATGG
	Reverse	TCTATCGGATACTTCAGCGTCA
TIMP1	Forward	CGAGACCACCTTATACCAGCG
	Reverse	ATGACTGGGGTGTAGGCGTA
MMP2	Forward	GGACAAGTGGTCCGCGTAAA
	Reverse	CCGACCGTTGAACAGGAAGG
TGF-β1	Forward	CCACCTGCAAGACCATCGAC
	Reverse	CTGGCGAGCCTTAGTTTGGAC
IκBα	Forward	GCCCCGCACAGCCATGTTTC
	Reverse	AGCGGACAGGCGAGGAGAGC
LC3	Forward	GACCGCTGTAAGGAGGTGC
	Reverse	AGAAGCCGAAGGTTTCTTGGG
β-actin	Forward	GGCTGTATTCCCCTCCATCG
	Reverse	CCAGTTGGTAACAATGCCATGT

### 4.10. Statistical Analysis

The positive areas of H & E, MT, and immunohistochemical staining were analyzed using Image-Pro Plus 6.0. All results are expressed as the mean ± standard deviation (SD). Comparisons between two groups were made using the Student’s *t*-test. Statistical differences in multiple groups were identified by analysis of variance (ANOVA), followed by the Tukey’s *post hoc* test. Statistical comparisons were made using SPSS 20.0 statistical analysis software (IBM-SPSS, Inc., Chicago, IL, USA). A probability *p* value of <0.05 was considered statistically significant.
